# Tibial deformities caused by focal fibrocartilaginous dysplasia: a narrative review

**DOI:** 10.3389/fped.2025.1584512

**Published:** 2025-05-14

**Authors:** Dimitri Fasel, Elio Paris, Victor Aye, Maxime Pilloux, Giacomo De Marco, Oscar Vazquez, Christina Steiger, Romain Dayer, Nathaly Gavira, Dimitri Ceroni

**Affiliations:** ^1^ Faculty of Medicine, University of Geneva, Geneva, Switzerland; ^2^ Pediatric Orthopedics Unit, Pediatric Surgery Service, Geneva University Hospitals, Geneva, Switzerland

**Keywords:** focal, fibrocartilaginous, dysplasia, tibia, metaphysis, sclerosis, varus, valgus

## Abstract

Focal fibrocartilaginous dysplasia (FFCD) of the tibia is a rare but well-recognised, “tumour-like” condition that primarily involves the proximal tibia and causes tibial deformities. Tibial FFCD affects infants and toddlers, and deformations are typically discovered when they begin to walk. The exact aetiology of FFCD remains unclear, even though several pathophysiological hypotheses have been proposed. It is thought that FFCD's natural course is towards spontaneous resolution within a few months or years, although there is occasionally some initial worsening before the correction begins. FFCD's radiographical appearance is so explicit and pathognomonic that no biopsy is required. Conservative management is considered the gold standard treatment for this condition. However, if the deformity worsens, persists over a longer period or is severe enough (greater than 30°), then surgical treatment may be indicated. This narrative review summarises more than 40 years of observations of patients with tibial FFCD, discusses its aetiology and revises information on its pathogenesis, clinical features, radiographical and histological characteristics, and treatment.

## 1 Introduction

Focal fibrocartilaginous dysplasia (FFCD) is an uncommon, benign condition of unknown aetiology that causes long-bone deformities in children. The vast majority of lesions involve the proximal tibia, and FFCD is typically associated with genu varum (commonly known as bow-leggedness). Since its original description by Bell et al. in 1985 (1), 105 cases of tibial FFCD have been described in the scientific literature (1–37). Usually diagnosed in infants and toddlers when they begin walking, FFCD is typically a self-limiting condition since most lesions resolve spontaneously, just as the tether likely broke spontaneously, and, thus, no treatment is required (17). However, if the deformity fails to resolve and progresses, surgical management may be required to correct it and prevent functional impairment. This narrative review summarises more than 40 years of observations of patients with tibial FFCD, discusses its aetiology and revises information on its pathogenesis, clinical features, radiographical and histological characteristics, and treatment. We hope to help paediatric orthopaedists establish a better, more informed care framework to optimise the management of this atypical bone disorder.

## 2 Historical review

In 1985, Bell et al. reported on a previously undescribed condition characterised by unilateral tibia vara associated with an area of FFCD in the proximal tibia's medial aspect (1). FFCD seems to have been a particularly well-chosen term for this bone anomaly, as it has remained unchanged for over 40 years. The authors of this first report were immediately interested in the condition's cause and developed a pathophysiological hypothesis that has never been refuted. Their explanation was that the mesenchymal anlage of the tibial metaphysis developed abnormally, for unknown reasons, at the insertion of the pes anserinus (1).

Since then, 56 other reports concerning FFCD have found their way into the literature, covering a total of 167 individual cases, of which 105 involved the tibia (1–37). The rest of this work takes data from these case reports and case series, the largest of which included 21 patients (23).

## 3 Epidemiology

As mentioned, 62.9% of reported cases of FFCD have been tibial, with 103 cases located at the proximal tibia and 2 at the distal tibia (1–37). At the level of the proximal tibia, 96.1% of lesions (99 cases) were located on the medial side of the metaphysis, resulting in a varus deformity (1–10, 12–25, 27–32, 34, 36, 37), whereas only 4 cases presented with damage to the lateral side of the metaphysis, resulting in a valgus deformity (11, 26, 33, 35). The two rare cases involving the distal tibia resulted in varus deformities (23, 26).

Tibial FFCD mainly affects infants, most of whom are under 4 years old at the time of diagnosis. Only one known case has involved an adult, and one might legitimately suggest that this was a residual deformity from FFCD that had occurred previously (28). The mean age of patients with tibial FFCD was 21.4 months old, ranging from 2 months to 29 years old (1–37); however, excluding the adult case, the mean age was 18.3 months. The sex distribution showed 46 females and 59 males (1–37). The lesion was systematically unilateral, affecting the right tibia in 51 cases, the left tibia in 49 and was unspecified in the remaining 4 cases (1–37). Finally, cases have been described among Caucasian, African and Asian patients, suggesting that the condition does not affect any one race selectively.

## 4 Aetiopathogenesis

The exact cause of FFCD and the mechanisms underlying its deformities are still not fully understood, and the subject is still a matter of debate. At first glance, the occurrence of deformities in very young children and the absence of any trauma or infection seem to argue for a congenital origin (6). In their original description of FFCD, Bell et al*.* postulated that it might result from the excessive production of fibrocartilage due to the abnormal differentiation of mesenchymal anlage in the area of the pes anserinus and the asymmetrical hampering of growth on one side of the proximal tibia (1). It has also been suggested that FFCD is an area of persistent fibrocartilaginous tissue adjacent to the growing plate, and it is suspected to act as a tether between the physis and the metaphysis, thereby leading to growth disturbance and angular deformity (26). Another report suggested that necrosis of the medial part of the physis was a predisposing condition, which was followed by regeneration and a diaphyseal defect (37). Other authors have attributed FFCD to displaced islets of the growth cartilage in the cortex of the juxtametaphyseal region (34). Other evidence led to suggestions that these injuries could have been caused by trauma during or after delivery, infections, or mechanical forces exerted on the tibia (1, 3, 9, 16, 17, 34, 38).

Due to the young age at which FFCD is diagnosed, the fact that it can occur after a trauma or an infection, and its propensity to correct itself spontaneously, the disorder has several troubling similarities with Cozen's phenomenon, also observed in the proximal tibia (39).

Other authors have proposed meaningful and somewhat different pathophysiological hypotheses, and we view Jouve et al*.*'s as the most likely. They suggested that FFCD should be considered as an abnormal anchorage of a tendon or a pathological fibrous band in the metaphyseal bone corresponding to a “fibrous periosteal inclusion” (17). The consequence is a disturbance to the periosteum's natural sliding along the diaphyseal bone during growth, inducing an epiphysiodesislike effect and similar consequences (17). The growth plate's unaffected portion continues to grow normally, leading to the aforementioned skeletal deformity. In the case of a varus deformity, the lesion is typically located at the pes anserinus tendon's insertion on the tibial metaphysis. In contrast, a valgus deformity is probably related to an anomaly of the fascia lata tensor tendon's insertion.

## 5 Clinical presentation & natural history

It remains extremely difficult to put a time or an age to the beginning of the processes that lead to FFCD. Indeed, since most patient histories emanate from case series or case reports, there are disparities in the timing and details of the data collected. The disorder's clinical course, therefore, appears to be far from uniform or even very inconsistent (21). The original case of FFCD reported that development had begun *in utero* (1), and the deformity has since then also been noted at birth (25). Nevertheless, some evidence suggests that FFCD could be caused by trauma during or after delivery, through infection or due to other mechanical forces exerted on the tibia (1, 3, 9, 17, 34, 38). As mentioned, Nakase et al*.* described a prodromal form of the affliction in a 2-month-old infant, characterised by a swelling of the leg with an important periosteal reaction in the medial aspect of the tibia's proximal metaphysis and associated with a subtle lesion of the bone at the same site. Since this discovery preceded the development of the tibial varus, the authors attributed the periosteal reaction with a role in inducing the deformity (27).

However, outward signs of FFCD are usually noticed by a child's parents shortly after the onset of standing and ambulation, positions in which the deformation appears much more easily apparent (17, 19). Patient age at the initial presentation of tibial FFCD varies from 2 months to 29 years old (1–37), but most patients express the disorder between 12 and 24 months old (6, 17). In our retrospective analysis of all the reported cases, the mean deformation described at the time of the lesion's discovery was 21.3° (1–37). Some lesions may initially be progressive, especially if the infant is very young. A progressive worsening of the deformity was documented in 40% of cases, and this can continue for up to 8 or 9 months (6). This increase in deformity can probably be attributed to the onset of weight-bearing and the local imbalance in force distribution (9). Subsequent resolution and correction typically start at around 21–24 months old (5, 6, 17, 22) and can continue for up to 26 months (to between 14 and 42 months) (5, 6, 17, 22). A spontaneous correction of the deformity occurred in 45% of reported cases (5, 8, 13, 14, 17, 22, 34), with the median time to recovery ranging between 57 and 65 months (6, 8). However, in most of the published case series, we noted that many patients had been operated on at an age at which one might have expected a spontaneous correction of the deformity to have already occurred. This suggests that the potential for spontaneous correction is probably even greater than our analysis of previous cases shows (6).

## 6 Radiological investigations

**Figure 1:**
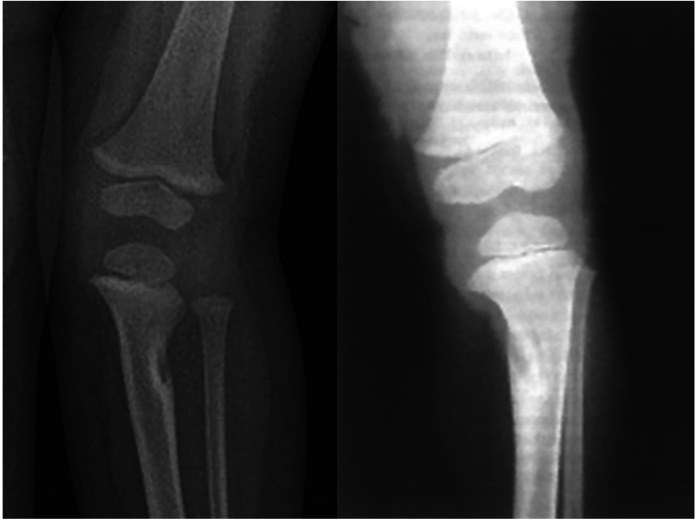
Two examples of FFCD; on the left, we can note an unusual valgus deformity in the left tibia characterised by a lateral defect surrounded by cortical condensation in its proximal third. On the right side, AP radiograph revealed a well-defined lucent defect in the medial cortex of the proximal tibia with varus deformity.

The typical radiographical pattern observed in tibial FFCD is a radiolucent cortical defect, usually located at the tibia's proximal and medial metaphysis, surrounded by a thick area of well-defined bone sclerosis (1). Much more rarely, the lesion is located on the lateral side of the tibia's metaphysis, and in such cases, a valgus deformity is apparent (11, 26, 33, 35). The characteristic finding on radiography images is an obliquely direct cortical gap located in the metaphyseal– diaphyseal area of the proximal third of the tibia (or, more exceptionally, in the distal third) (7). This gap does not appear well-defined within the concavity of the deformation, where the bone margin is usually absent (13, 17, 40). Varus or valgus deformity occurs and progresses at the cortical gap (6) (Figure 1). A prominent periosteal reaction has been described, although extremely rarely (13, 27). This can be disturbing and can affect the interpretation of the x-ray image; however, over time, the phenomenon regresses and dies away at the same time as the deformation appears. Indeed, some authors have evaluated that the cortical defect disappeared after a mean of 34 months (10).

The signs observed on conventional radiography images are typically clear enough to eliminate the need for more technologically advanced radiological investigations. Thus, most authors do not currently recommend routine MRI of this condition, but it may be justified for investigating an FFCD with an atypical clinical presentation (25, 34, 41). This is important because the children with this condition are infants or toddlers, and further investigation, including MRI, may require general anaesthesia. MRI findings are characterised by hypointense signals on T1-weighted sequences and hypointense or heterogenous signals on T2-weighted sequences (25). Low-intensity signals come from the lesion's margins and the fibrodysplasia itself, whereas high-intensity signals are due to the cartilage (25, 42, 43). Lou et al*.* suggested using T1-weighted, three-dimensional (3-D) VIBE MRI with a water excitation sequence since it improves the contrast between cartilage and other tissue components, more precisely highlighting the shape, thickness and enthesis of the fibrous band (23).

## 7 Histopathological investigations

Histopathological findings regarding FFCD lesions have included purely fibrous (8, 25, 29, 40) lesions with a strong fibrocartilaginous component (1, 3, 5, 8, 44, 17, 31, 15, 23) and even islands of hyaline cartilage (8). Kim et al*.* reported that the results of histopathological examinations could differ significantly from one patient to another, suggesting that the condition was evolutionary and underwent maturational changes from an initial cellular condition to a cartilaginous stage, on to a late paucicellular phase and finally a more fibrous stage (23). Under the microscope, they also observed that there was a transition of tissue types moving into the lesion, from cartilage tissue or fibrocartilage tissue towards purely fibrous tissue, suggesting that FFCD lesions were closely related to epiphyseal cartilage (23). Given FFCD's broad and blurred spectrum of histological features— resulting in the fact that a definitive distinction between FFCD and the fibrous tether remains elusive—Kim et al*.* even suggested naming this lesion a “subperiosteal fibrocartilaginous pseudotumor of long bones” (20).

## 8 Treatment

The management of tibial FFCD has changed significantly over the last 40 years as knowledge about it has improved. Initially, many cases of FFCD were treated surgically, primarily to establish a histological diagnosis and confirm the benign nature of the lesion (5, 23, 37). Most of the time, these biopsies were coupled with curettage of the lesion, which probably included periosteal release. Since it is recognised that radiography images of FFCD exhibit quite distinctive characteristics, the indication for performing a biopsy is no longer justifiable (20).

Given the extreme disparity in the measures used to treat these cases, it is currently very difficult to draw a coherent set of guidelines regarding the indications for surgical treatment. Indeed, surgical treatment can be the response to very disparate indications; however, some of these do not stand up to scientific scrutiny. Firstly, many biopsies have been and still are proposed to ensure a diagnostic confirmation of FFCD, to reassure both the family and their doctors (19). In many cases, families feel the need to push for surgical treatment because of their grave concerns about the possible effects of the limb deformity on their child's gait, physical development (26) and long-term psychological development (23). Families very often even feel a need for surgery to hasten the self-correction process (26).

The angular cut-off value at which surgery becomes legitimate is a point of disagreement. Some authors consider that tibial FFCD does not require treatment if the Levine and Drennan angle is less than 30°, recommending that children be treated conservatively or kept under observation for 24 months before any surgical intervention (31). This wait-and-see attitude is most likely based on the recognition and acceptance that an infantile growth plate is able to correct a tibial deformity due to FFCD if it is less than 30° (36). Other authors have set much lower cut-off angles for tibia deformity, setting a 20° limit for the Levine and Drennan angle and suggesting a suitable period of observation of 6–12 months (8, 17).

However, both sets of authors suggested that curettage should be considered when a child around 24 months old has a Levine and Drennan angle greater than 20° and this widens within a 6–12 month observation window (8, 17).

Several operative strategies have been described, all with seemingly equivalent results. Indeed, the evidence seems to demonstrate that the majority of children who have undergone surgical treatment—including procedures such as fibrous band release, periosteal release, curettage, osteotomy, guided growth or combinations of different surgical techniques—have achieved clinical and radiological healing by the time of their last scheduled follow-up consultation (3, 17). Thus, it is up to the attending surgeon to select the least invasive surgical solution that will provide a satisfactory clinical result. Curettage is considered a minimally invasive procedure with low complication rates, and it seems to be very effective at stimulating the deformity's correction. Simple curettage has proved to be very effective, inducing a mean change in angle of 2° per month (17). Choi et al*.* even reported a very effective post-curettage correction rate of greater than 2° per month (8). Curettage seems to be very powerful because it probably removes the fibrous tether, and releasing this brake stimulates growth (21).

Curettage should, therefore, be preferred to osteotomies, which have their share of complications (17), such as peroneal nerve palsy and the onset of valgus deformity due to Cozen's phenomenon (3, 5). Corrective osteotomy seems to have been reserved for children with more severe tibial deformities, especially a Levine and Drennan angle significantly greater than 30° (3, 5, 17). Some authors have also suggested correcting angular deformities due to FFCD of the tibia by using guided growth (2, 21, 26). However, we should remember some points that could modulate enthusiasm for this technique. Firstly, it is important to understand that this technique in no way releases the brake on growth that sits on the side contralateral to the hemiepiphysiodesis. Secondly, corrective osteotomy does not correct the deformation's centre of rotation. Thirdly, performing a hemiepiphysiodesis on a child younger than 4 years old can be technically difficult, and not without risk, due to the immaturity of the proximal tibia's chondrepiphysis at this age (2).

It is important to emphasise that our analysis of the cases that had been operated on showed a mean age at the time of surgery of 25.2 months old, and the average angle of the deformity was 23.8°, suggesting that surgery was performed at ages and for deformities that still had the potential to correct spontaneously (1–37).

## 9 Conclusion

Focal fibrocartilaginous dysplasia (FFCD) of the tibia is a rare benign condition characterised by its spontaneous resolution. It mainly involves the proximal tibia and likely causes angular deformities that generally worsen up to the age of 20–24 months old. The lesion has usually corrected itself completely by the child's 65th month. FFCD's radiographical appearance is sufficiently explicit to negate any indication for magnetic resonance imaging or a biopsy. A monitoring policy should be implemented, and only a worsening deformity after 3 years old should justify exceptional recourse to a surgical correction. When this is required, curettage of the lesion seems to be sufficient to achieve a progressive correction of the deformity, at a rate of around 2° per month. Curettage of the lesion seems to be the most appropriate procedure and should, therefore, be performed before any thought of osteotomy, which seems to be burdened by a higher rate of complications.
